# FXYD6: a novel therapeutic target toward hepatocellular carcinoma

**DOI:** 10.1007/s13238-014-0045-0

**Published:** 2014-04-10

**Authors:** Qian Gao, Xiongfei Chen, Hongxia Duan, Zhaoqing Wang, Jing Feng, Dongling Yang, Lina Song, Ningxin Zhou, Xiyun Yan

**Affiliations:** 1 Key Laboratory of Protein and Peptide Pharmaceuticals, CAS-University of Tokyo Joint Laboratory of Structural Virology and Immunology, Institute of Biophysics, Chinese Academy of Sciences, Beijing, 100101 China; 2 University of Chinese Academy of Sciences, Beijing, 100049 China; 3 Medical College of Soochow University, Industrial Park, Suzhou, 215123 China; 4 Institute of Hepatobiliary Gastrointestinal Disease, General Hospital of PLA Second Artillery, Beijing, 100088 China

**Keywords:** FXYD6, hepatocellular carcinoma (HCC), tumor progression, therapeutic target, Na^+^/K^+^-ATPase, Src-ERK signaling pathway

## Abstract

FXYD6, FXYD domain containing ion transport regulator 6, has been reported to affect the activity of Na^+^/K^+^-ATPase and be associated with mental diseases. Here, we demonstrate that FXYD6 is up-regulated in hepatocellular carcinoma (HCC) and enhances the migration and proliferation of HCC cells. Up-regulation of FXYD6 not only positively correlates with the increase of Na^+^/K^+^-ATPase but also coordinates with the activation of its downstream Src-ERK signaling pathway. More importantly, blocking FXYD6 by its functional antibody significantly inhibits the growth potential of the xenografted HCC tumors in mice, indicating that FXYD6 represents a potential therapeutic target toward HCC. Altogether, our results establish a critical role of FXYD6 in HCC progression and suggest that the therapy targeting FXYD6 can benefit the clinical treatment toward HCC patients.

**Electronic supplementary material:**

The online version of this article (doi:10.1007/s13238-014-0045-0) contains supplementary material, which is available to authorized users.

## Introduction

FXYD6 (FXYD domain containing ion transport regulator 6) is the sixth defined member of the FXYD family, whose members are named due to the presence of a highly conserved FXYD (Phe-X-Tyr-Asp; X, any amino acid) motif in their amino acid sequences (Sweadner and Rael, 2000). Mammalian FXYD family consists of seven members of FXYD1–7, which are type I trans-membrane proteins and are similar to each other not only in their protein structure but also in their function (Geering, 2006).

FXYDs are regulators of Na^+^/K^+^-ATPase, which is located on the cellular membrane and is composed of α and β subunits. FXYDs modulate the holo-enzyme kinetic properties of Na^+^/K^+^-ATPase by changing the rate and affinity of Na^+^ and K^+^ transport (Garty and Karlish, 2006). Recent report indicated that FXYDs stabilize the active conformation of the Na^+^/K^+^-ATPase by direct interaction with this enzyme (Mishra et al., 2011). It is noteworthy that, besides its major role as the Na^+^/K^+^ pumps, Na^+^/K^+^-ATPase is involved in tumor proliferation and migration through activating its downstream Src-ERK signaling components (Prassas and Diamandis, 2008). The involvement of the holo-enzyme of Na^+^/K^+^-ATPase in tumor progression is further manifested by the high abundance of its  α1 subunits in clinical HCC samples (Xu et al., 2010).

As the critical regulators of Na^+^/K^+^-ATPase, the relevance of FXYDs to tumors recently has also been attracting a plethora of attention. It has been reported that FXYD2 (Gaut et al., 2013), FXYD3 (Kayed et al., 2006; Widegren et al., 2009) and FXYD5 (Sato et al., 2003) are up-regulated in several types of tumors, leading to accelerated tumor growth and progression (Grzmil et al., 2004; Ino et al., 2002).

However, whether or not FXYD6 is up-regulated and involved in tumor progression, particularly in HCC, remains elusive. We find that FXYD6 is up-regulated in HCC and its expression levels are positively associated with the increased migration potential and proliferation rate in HCC cells. Importantly, blockade of FXYD6 by our self-generated anti-FXYD6 functional antibody significantly inhibited xenografted tumor growth, suggesting that FXYD6 is a novel therapeutic target toward HCC.

## Results

### Anti-FXYD6 antibody of FD10 possesses high specificity and affinity

Except minor investigations showing the FXYD6 transcript was detected in human central nervous system and associated with some mental disorders (Choudhury et al., 2007; Shiina et al., 2010), FXYD6 expression profile in other tissues, especially in tumor tissues, remains largly unknown. To determine the role of FXYD6 in tumor progression, we firstly generated a panel of mouse mAbs (monoclonal antibody). According to the enzyme-linked immunosorbent assay (ELISA) results, the FD10 mAb against FXYD6 was selected for further performance evaluation.

**Figure 1:**
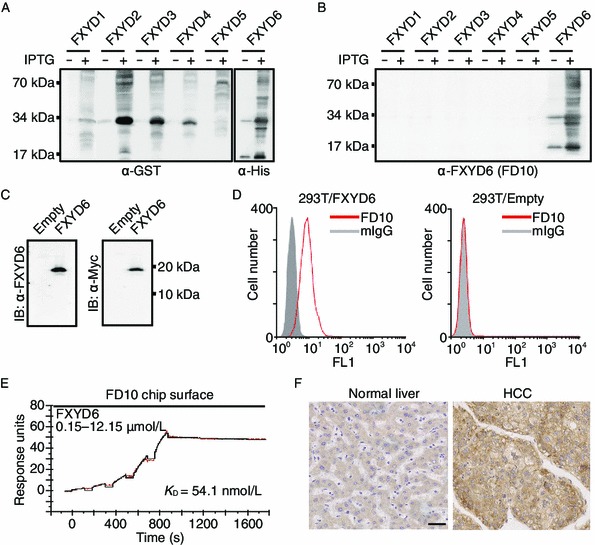
**Anti-FXYD6 mAb of FD10 possesses high specificity and affinity**. (A and B) Bacteria-expressed FXYD1–6 proteins from whole cell lysate without or with IPTG were detected by anti-Tag (A) or anti-FXYD6 mAb of FD10 (B). (C) Immunoblotting analysis of FXYD6 expression in 293T cells. Cells were transfected with pcDNA3.1-empty or pcDNA3.1-FXYD6, and whole cell lysate was analyzed using anti-FXYD6 antibody of FD10 (left panel) or anti-myc antibody (right panel). (D) FACS analysis of FD10 binding with FXYD6. The 293T cells, transfected with pcDNA3.1-FXYD6 (left panel) or with pcDNA3.1-empty (right panel), were incubated with FD10 or control normal IgG. (E) SPR sensorgrams illustrating binding of FD10 to FXYD6 proteins (black line) and fitted saturation binding curves (red line). The dissociation constant (*K*_*D*_) was calculated from the saturation binding curve. (F) Normal liver tissue (left panel) and HCC tissue (right panel) were stained by immunohistochemistry with FD10 (bar, 40 μm)

To test the sole specificity of FD10 to its own antigen of FXYD6, we produced the exogenously expressed FXYD1 to FXYD5 proteins by inserting the fragments containing the FXYD1–5 cDNA coding sequences into the prokaryotic expression vector pGEX-6P-1-GST to produce the plasmids of pGEX-6P-1-GST-FXYD1–5. Meanwhile, we constructed the plasmid of pET28a-His-FXYD6 to be used as a positive control for immunoblotting. The correct expression of GST-tagged FXYD1 to FXYD5 and the His-tagged FXYD6 was confirmed using anti-GST and anti-His antibody, respectively (Fig. 1A). FD10 only recognized FXYD6, but not FXYD 1–5 (Fig. 1B). Such specificity was further confirmed by immunoblotting with eukaryotic FXYD6 proteins, in which FD10 specifically detected a single band of approximately 20 kDa under reducing conditions in the 293T cells transfectants of pcDNA3.1-FXYD6 (Fig. 1C, left panel). The in-frame correct expression of this Myc-tagged FXYD6 was further confirmed using the commercial Myc-Tag mAb (Fig. 1C, right panel).

Fluorescence activated cell sorting (FACS) analysis showed that FD10 bound with cells harboring pcDNA3.1-FXYD6 (Fig. 1D, left panel), whereas no such binding was detected in cells with pcDNA3.1-empty vector only (Fig. 1D, right panel), demonstrating that FD10 could recognize the FXYD6 with native conformation. Surface plasmon resonance (SPR) assay data showed that the equilibrium dissociation constant (*K*_*D*_) of FD10 to FXYD6 was 54.1 nmol/L (Fig. 1E), indicating that the affinity of FD10 to its antigen of FXYD6 is relatively high. Taken together, these results show that our self-generated FD10 can be used in further investigation of the FXYD6’s role in tumor progression.

### FXYD6 is up-regulated in some types of human tumors

To clarify whether or not the difference of FXYD6 protein levels is existed between normal versus cancerous tissues, using FD10, we conducted immunohistochemical screen on a commercial human tissue array with a total of 397 samples (Table S1). FXYD6 was screened out from a large proportion of tumor samples and the expression profile of FXYD6 was summarized in Table 1. Although no significant difference was found on FXYD6 levels between the cancerous and the normal tissues in the following tested tumors including cerebrum, uterus, bladder, ovary, breast, testis, lung, stomach and pancreas. The significant high levels of FXYD6 were detected in tumors of liver, thyroid, prostate and colon, compared with the corresponding normal tissues (Fig. 1F). HCC and normal liver tissues accounted for the largest proportion in the detected samples, implying that FXYD6 may play a critical role in the progression of HCC.

**Table 1:** The expression profile of FXYD6 in human tissues

Type of tissues		Positive/Total	Positive (%)	*P*-value
Liver	Hepatocellular carcinoma	54/56	96	*P* < 0.01
	Normal tissue	11/35	31	
Thyroid	Carcinoma	17/17	100	*P* < 0.01
	Normal tissue	2/6	33	
Prostate	Adenocarcinoma	9/13	69	0.01 < *P* < 0.05
	Normal tissue	1/6	17	
Colon	Adenocarcinoma	17/17	100	0.01 < *P* < 0.05
	Normal tissue	4/6	67	
Cerebrum	Astrocytoma	17/17	100	NS
	Normal tissue	7/7	100	
Uterus	Adenocarcinoma	20/20	100	NS
	Normal tissue	5/5	100	
Bladder	Transitional cell carcinoma	18/20	90	NS
	Normal tissue	1/1	100	
Ovary	Carcinoma	20/30	67	NS
	Normal tissue	4/6	67	
Breast	Carcinoma	18/29	62	NS
	Normal tissue	6/9	67	
Testis	Seminoma	12/21	57	NS
	Normal tissue	5/6	83	
Lung	Carcinoma	14/25	56	NS
	Normal tissue	1/1	100	
Stomach	Adenocarcinoma	9/18	50	NS
	Normal tissue	3/5	60	
Pancreas	Adenocarcinoma	5/15	33	NS
	Normal tissue	3/6	50	

NS: not significant

### FXYD6 promotes migration and proliferation of HCC cells

**Figure 2:**
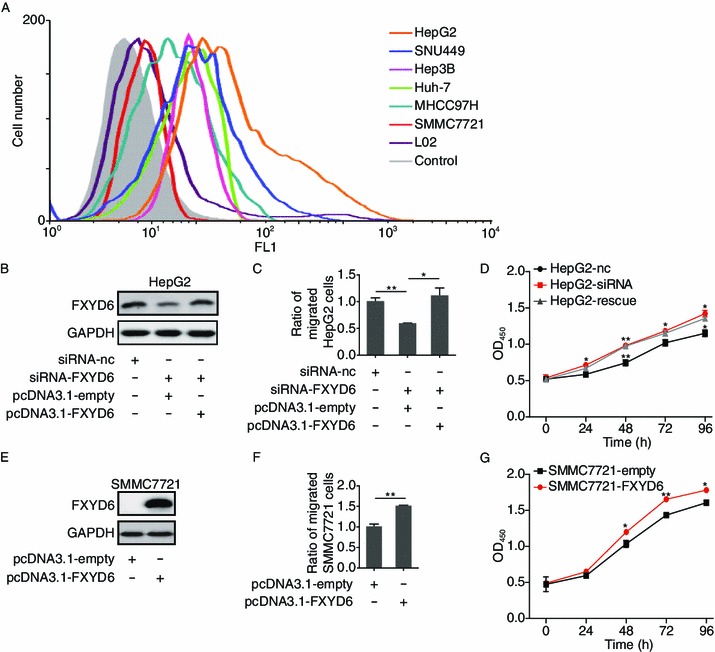
**FXYD6 promotes the migration and proliferation of HCC cells**. (A) FACS analyzed the expression of FXYD6 protein within 6 HCC cell lines and L02 control cell line using FD10. Control cells were SMMC7721 stained by mIgG. (B) Immunoblotting of FXYD6 protein in HepG2 cells after 36 h co-transfection with siRNA-FXYD6 and pcDNA3.1-FXYD6. (C) The migration of HepG2 cells was determined after 36 h co-transfection with siRNA-FXYD6 and pcDNA3.1-FXYD6. *P < 0.05 or **P < 0.01, compared with the siRNA-FXYD6 group. (D) The proliferation of HepG2 cells was determined after co-transfection with siRNA-FXYD6 and pcDNA3.1-FXYD6 at different time points. Rescue assay was conducted in HepG2 cells co-transfected with siRNA-FXYD6 and pcDNA3.1-FXYD6. *P < 0.05 or **P < 0.01, compared with the siRNA-FXYD6 group. (E) Immunoblotting of FXYD6 protein in SMMC7721 cells after 24 h transfection with pcDNA3.1-FXYD6. (F) The migration of SMMC7721 cells was determined after 24 h transfection with pcDNA3.1-FXYD6. **P < 0.01, compared with the pcDNA3.1-empty group. (G) The proliferation of SMMC7721 cells was determined after transfection with pcDNA3.1-FXYD6 at different time points. *P < 0.05 or **P < 0.01, compared with the pcDNA3.1-empty group. Data were collected from 3 independent experiments and the number of migrated cells from the controls was normalized as one

To further explore the implication of FXYD6 up-regulation in HCC progression, we collected six HCC cell lines including HepG2, SNU449, Hep3B, Huh-7, MHCC97H and SMMC7721 for this investigation. Meanwhile, the L02 cell was included as the normal control for liver cell line. Consistent with the immunohistochemical results (Table 1), FACS data showed that FXYD6 was detected out from almost all tested HCC cell lines but not from L02 cells. Among these 6 HCC cell lines, the cell line harboring the highest abundance of FXYD6 was HepG2, while the cell line with lowest FXYD6 was SMMC7721 (Fig. 2A). Therefore, the FXYD6-highest HepG2 and the FXYD6-lowest SMMC7721 cells were ideal cell lines for observation of the correlation between the FXYD abundance and tumor progression.

Knocking down of FXYD6 by siRNA in HepG2 cells (Fig. 2B) not only inhibited cell migration but also decreased cell proliferation, and such inhibition was restored when the down-regulated FXYD6 was rescued by exogenous over-expression of FXYD6 (Fig. 2C and 2D). Conversely, enforced over-expression of FXYD6 in SMMC7721 cells (Fig. 2E) increased migration and proliferation of these cells, compared with control transfectants of empty vector (Fig. 2F and 2G). Therefore, these results demonstrate that the FXYD6 expression levels are positively correlated with HCC cells migration and proliferation, suggesting that FXYD6 is implicated in tumor progression of HCC.

### FXYD6 is associated with the Na^+^/K^+^-ATPase in HCC cells

**Figure 3:**
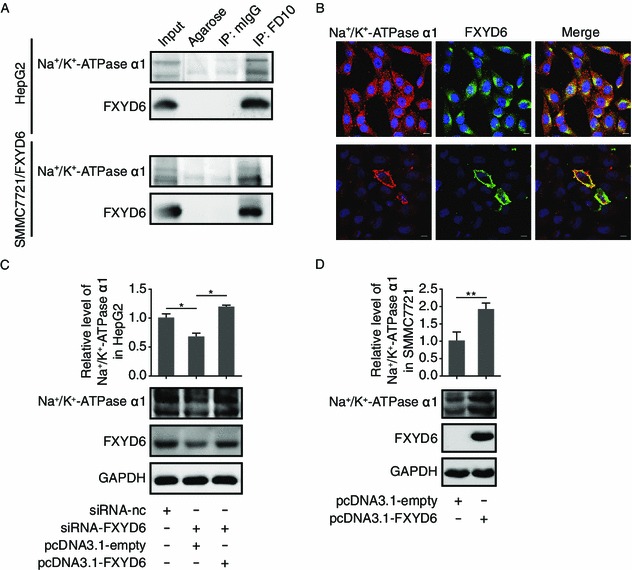
**FXYD6 associates with Na**^**+**^**/K**^**+**^**-ATPase in HCC**. (A) Co-IP analysis of the interaction between FXYD6 and Na^+^/K^+^-ATPase α1 in HepG2 cells (upper panel) and in SMMC7721 cells transfected with pcDNA3.1-FXYD6 (lower panel). The immunoprecipitates were analyzed by immunoblotting with anti-Na^+^/K^+^-ATPase α1 and FD10 antibodies. (B) Co-localization analysis of FXYD6 and Na^+^/K^+^-ATPase α1 in same cell lines as described in (A) using double immunostaining with FD10 (green) and anti-Na^+^/K^+^-ATPase α1 (red). Nuclei were stained with DAPI (blue) and colocalization is indicated in yellow (bar, 10 μm). (C and D) Immunoblotting of Na^+^/K^+^-ATPase α1 subunit expression in HepG2 cells (C) after 48 h co-transfection with siRNA-FXYD6 and pcDNA3.1-FXYD6 or in SMMC7721 cells (D) after 48 h transfection with pcDNA3.1-FXYD6. Immunoblotting data from at least 3 independent experiments were measured with the band density, which were normalized to GAPDH. The signals from control cells were put to one. The bar graphs (mean ± SEM) and representative images are shown. *P < 0.05 or **P < 0.01, compared with the control

Although FXYD6 has been known to be a regulator of Na^+^/K^+^-ATPase and Delprat et al. has revealed the relation of FXYD6 with Na^+^/K^+^-ATPase in rat inner ear (Delprat et al., 2007a; Delprat et al., 2007b), whether or not FXYD6 interacts with the Na^+^/K^+^-ATPase in HCC remains elusive. Co-immunoprecitation (co-IP) results indicated that the interaction existed between FXYD6 and Na^+^/K^+^-ATPase not only in HepG2 cells (Fig. 3A, upper panel) but also in SMMC7721 cells harboring FXYD6 expressing vector (Fig. 3A, lower panel). This interaction was further confirmed by immunofluorescence assay, by which we observed the co-localization of FXYD6 and Na^+^/K^+^-ATPase in the HCC tissues (Fig. S1), in HepG2 (Fig. 3B, upper panel) and in SMMC7721 transfectants of FXYD6 (Fig. 3B, lower panel). More interestingly, we observed that the increased expression levels of FXYD6 were positively correlated with the enhanced protein levels of Na^+^/K^+^-ATPase α1 (Fig. 3C and 3D), indicating that FXYD6 exerts its regulatory effect on the holo-enzymatic activity of Na^+^/K^+^-ATPase through up-regulating the protein levels of Na^+^/K^+^-ATPase α1 besides the direct interaction with this enzyme.

### FXYD6 promotes the activation of Na^+^/K^+^-ATPase downstream signaling

**Figure 4:**
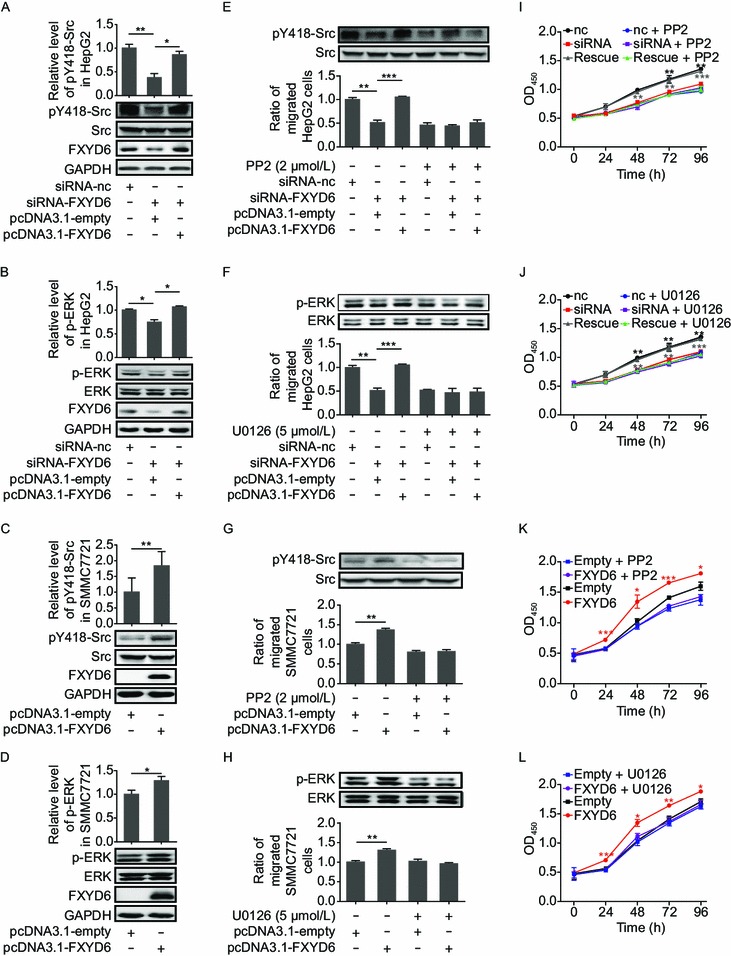
**FXYD6 activates Src and ERK signaling pathways in HCC cells**. (A and B) The phosphorylations of Y418-Src (A) and ERK (B) were determined in HepG2 cells co-tranfected with siRNA-FXYD6 and pcDNA3.1-FXYD6. *P < 0.05 or **P < 0.01, compared with the siRNA-FXYD6 group. (C and D) The phosphorylations of Y418-Src (C) and ERK (D) were determined in SMMC7721 cells transfected with pcDNA3.1-FXYD6. *P < 0.05 or **P < 0.01, compared with the pcDNA3.1-empty group. The band density (mean ± SEM) was measured from at least 3 independent immunoblots and was normalized to the paired total. The signal from control cells was put to one. The representative images are shown. (E and F) The phosphorylation of Y418-Src in the presence of Src inhibitor PP2 (2 μmol/L) (E, upper panel) or the phosphorylation of ERK in presence of ERK inhibitor U0126 (5 μmol/L) (F, upper panel) was determined by immunoblotting and the migration (lower panel) of HepG2 cells was determined after co-transfection with siRNA-FXYD6 and pcDNA3.1-FXYD6. **P < 0.01 or ***P < 0.001, compared with the siRNA-FXYD6 alone group. (G and H) The phosphorylation of Y418-Src in the presence of Src inhibitor PP2 (2 μmol/L) (G, upper panel) or the phosphorylation of ERK in the presence of ERK inhibitor U0126 (5 μmol/L) (H, upper panel) was determined by immunoblotting and the migration (lower panel) was determined in SMMC7721 cells transfected with pcDNA3.1-FXYD6. **P < 0.01, compared with the pcDNA3.1-empty alone group. (I and J) The proliferation of HepG2 cells was determined after co-transfection with siRNA-FXYD6 and pcDNA3.1-FXYD6 in the presence of Src inhibitor PP2 (2 μmol/L) (I) or ERK inhibitor U0126 (5 μmol/L) (J) at different time points. **P < 0.01 or ***P < 0.001, compared with the siRNA-FXYD6 alone group. Rescue assay was conducted in HepG2 cells co-transfected with siRNA-FXYD6 and pcDNA3.1-FXYD6. (K and L) The proliferation was determined in SMMC7721 cells transfected with pcDNA3.1-FXYD6 in the presence of Src inhibitor PP2 (2 μmol/L) (K) or ERK inhibitor U0126 (5 μmol/L) (L) at different time points. *P < 0.05, **P < 0.01, or ***P < 0.001, compared with the pcDNA3.1-empty alone group. Data were collected from 3 independent experiments and the number of migrated cells from the controls was normalized as one

Some oncogenic signaling pathways, such as the Src and ERK pathways, are tightly associated with Na^+^/K^+^-ATPase-mediated tumor cells migration and proliferation (Prassas and Diamandis, 2008). To determine the mechanism underlying FXYD6-induced tumor cell migration and proliferation, we measured the phosphorylation of both kinases when FXYD6 expression levels were modulated. In HepG2 cells, knocking down of FXYD6 by siRNA significantly decreased the phosphorylation of Src and ERK (Fig. 4A and 4B); such decrease was rescued when FXYD6 was restored by exogenously expressing FXYD6. In contrast, the levels of the phosphorylated Src and ERK in the SMMC7721 cells were dramatically increased after transfection with FXYD6-containing vector compared with control transfectants (Fig. 4C and 4D). In HepG2 cells, such positive correlation between FXYD6 protein levels and the Src or ERK phosphorylation levels (Fig. 4E and 4F, upper in columns 1–3) disappeared when the specific Src inhibitor PP2 or ERK inhibitor U0126 was added, respectively (Fig. 4E and 4F, upper in columns 4–6). Consistently, in SMMC7721 cells, such correlation was also not detected in the presence of PP2 or U0126 (Fig. 4G and 4H, upper panel).

Consequently, in the presence of PP2 or U0126, down-regulation of FXYD6 by siRNA no longer had significantly inhibitory effect on the migration (Fig. 4E and 4F, lower panel) or proliferation (Fig. 4I and 4J) of HepG2 cells. Conversely, in SMMC7721 cells, up-regulation of FXYD6 by over-expression had no more enhanced effects on the migration (Fig. 4G and 4H, lower panel) and proliferation (Fig. 4K and 4L) when the Src inhibitor PP2 or the ERK inhibitor U0126 was applied, respectively. These data together demonstrate that the enhancement of FXYD6 is closely related with the increased migration and proliferation of HCC cells, which is dependent on the activation of Src or ERK, the downstream signaling components of Na^+^/K^+^-ATPase. Therefore, our data provide the mechanism insight in FXYD6-promoted HCC progression.

### Blocking FXYD6 with its antibody inhibits liver tumor growth

**Figure 5:**
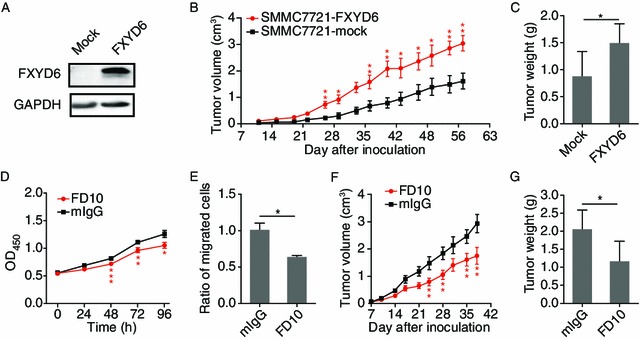
**FXYD6 promotes tumor growth*in vivo***. (A) FXYD6 expression in stable SMMC7721-mock and SMMC7721-FXYD6 cell lines. (B) Mean tumor volumes were measured at indicated time points after mice injection with SMMC7721-mock cells or SMMC7721-FXYD6 cells (*n* = 6). (C) When tumor size reached to approximately 3 cm^3^, mice were sacrificed and tumors were excised and the weight was evaluated. (D and E) The proliferation (D) and migration (E) of HepG2 cells were determined in the presence of FD10 or mIgG. (F and G) HepG2 cells were injected subcutaneously into nude mice. After forming tumor, mice were treated with FD10 and mIgG twice a week (*n* = 5). Tumor volume was calculated (F) and the weight was measured after sacrifice (G). *P < 0.05, **P < 0.01, or ***P < 0.001, compared with the control

To assess whether FXYD6 was involved in the tumor formation and growth *in vivo*, we established xenografted HCC models in nude mice using the stable transfectants of SMMC7721-mock and SMMC7721-FXYD6 cells (Fig. 5A). Both cell lines were able to form tumors; however, the tumors originated from SMMC7721-FXYD6 cells owned larger volume and heavier weight than both indexes from SMMC7721-mock cells (Fig. 5B and 5C). Thus, FXYD6 promoting tumor growth suggests FXYD6 may be a therapeutic target for HCC.

To test whether or not the anti-FXYD6 mAb FD10 had blocking effects on FXYD6 function, FXYD6-highest HepG2 cells were used for this *in vitro* examination. In HepG2 cells, the proliferation (Fig. 5D) and migration (Fig. 5E) were significantly inhibited in the presence of FD10 compared with control antibody (mIgG) incubation. Next, we established HepG2 cells xenografted tumor model in nude mice to examine the effect of FD10 on tumor therapy *in vivo*. FD10 or mIgG treatment was started when tumors reached a volume of 0.5–0.6 cm diameter. In the FD10-treated group, the significant reduction of tumor volume (Fig. 5F) and weight (Fig. 5G) was observed compared with that in the mIgG treated control group. Taken together, these data suggest that FXYD6 is a novel and potential therapeutic target toward HCC and that therapy against FXYD6 is an effective strategy toward HCC.

## DISCUSSION

This study for the first time reveals that FXYD6 protein expression levels are enhanced in HCC. We find that overexpression of FXYD6 endowed HCC cells with the advantage of migration and growth. Importantly, we show that the mechanism by which increase of FXYD6 enhances HCC cells’ migration and growth is attributable to activation of Na^+^/K^+^-ATPase down-stream signaling pathway. Furthermore, we show that blocking FXYD6 with its mAb inhibits liver tumor growth. Thus, these findings indicate that FXYD6 is a potential therapeutic target to attenuate HCC malignant characteristics.

To assess FXYD6 protein expression profile in human tissues, especially in cancerous tissues, the specific anti-FXYD6 antibody is urgently needed. To this end, we generated the anti-FXYD6 antibody of FD10, who possesses high specificity and affinity with FXYD6 (Fig. 1). With this powerful tool, we provide the first evidence for human FXYD6 protein expression profile. Consistent with the reported mRNA expression pattern of FXYD6 in brain (Choudhury et al., 2007; Shiina et al., 2010), FXYD6 protein levels were also high in cerebrum tissues. Additionally, we found that, compared with the normal counterparts, FXYD6 protein abundance was significantly higher in some types of cancerous tissues, suggesting FXYD6 is likely a novel biomarker for these tumors (Table 1).

Inspired by the close correlation between FXYD3/FXYD5 and tumor progression (Kayed et al., 2006; Sato et al., 2003; Widegren et al., 2009), we supposed that FXYD6 may be also involved in tumor malignancy. We have found that knockdown of FXYD6 by siRNA decreases migration (Fig. 2C) and proliferation (Fig. 2D) in HepG2 cells. Furthermore, we have found that overexpression of FXYD6 increases migration and proliferation in the human SMMC7721 cells (Fig. 2F and 2G), which agrees with the role of FXYD3 or FXYD5 overexpression in tumor progression. Thus, these observations establish that FXYD6 plays an important role in HCC progression.

Na^+^/K^+^-ATPase α subunits are new targets in anticancer therapy (Mijatovic et al., 2008). Delprat et al. has revealed the direct interaction of FXYD6 with Na^+^/K^+^-ATPase α1 subunit in rat inner ear (Delprat et al., 2007a; Delprat et al., 2007b). Consistently, in HCC cells, such interaction was also detected, indicating that this interaction is not cell type specific (Fig. 3A and 3B). In addition, we have found that FXYD6 induces the expression of Na^+^/K^+^-ATPase α1 subunit (Fig. 3C and 3D), which may account for the up-regulation of Na^+^/K^+^-ATPase α1 in HCC tissues as described in the previous report (Xu et al., 2010).

Na^+^/K^+^-ATPase is involved in tumor progression through activation of its down-stream Src-ERK signaling pathway (Prassas and Diamandis, 2008). We have found that knockdown of FXYD6 by siRNA decreases the activation of Src (Fig. 4A) and ERK (Fig. 4B) in HepG2 cells. Conversely, overexpression of FXYD6 increases the activation of Src (Fig. 4C) and ERK (Fig. 4D) in the human SMMC7721 cells. In addition, when the Src or ERK is inhibited by their specific inhibitors, the positive correlation between the FXYD abundance and HCC cells survival advantage of migration and proliferation is abolished (Fig. 4E–L). Therefore, these observations indicate that the role of FXYD6 in promoting HCC progression is through activating the down-stream pro-survival Src-ERK signaling pathway of Na^+^/K^+^-ATPase. Consistent with the function of FXYD6 in endowing HCC cells survival advantage (Fig. 2) *in vitro*, we have also shown that blockade of FXYD6 activity by its functional antibody can significantly reduce the volume and weight of xenografted tumors originated from HCC cells *in vivo* (Fig. 5), suggesting that FXYD6 is an important mediator in tumor development.

In conclusion, we provide the evidence that FXYD6 is a novel biomarker for tumors of liver, thyroid, prostate and colon. The up-regulation of FXYD6 is coordinated with the increase of Na^+^/K^+^-ATPase α1 subunit as well as with the activation of Na^+^/K^+^-ATPase signaling pathway in HCC. Importantly, we showed that blockade of FXYD6 by its functional antibody generated by our laboratory significantly inhibited tumor growth *in vivo.* Thus, we present the first insight of FXYD6-mediated tumor progression and speculate that anti-FXYD6 therapy may be an effective strategy toward HCC treatment.

## MATERIALS AND METHODS

### Construction of plasmids

The plasmids of pGEX-6P-1-GST-FXYD1 to pGEX-6P-1-GST-FXYD5, pET28a-His-FXYD6 and pcDNA3.1-myc-FXYD6 were generated by inserting the corresponding full length cDNAs into the empty vectors.

### Generation of anti-FXYD6 antibody of FD10

The recombinant FXYD6 proteins antigen was produced by bacteria, and purified from the soluble cell lysate fractions by nickel affinity chromatography. The anti-human FXYD6 mAb of FD10 was generated from mouse. The mAb was purified from mice ascites and the isotype was IgG2 determined by a mouse monoclonal antibody isotyping kit (Sigma) according to the manufacturer’s instructions.

### Animals

BALB/c nude mice were obtained from the Animal Center of the Chinese Academy of Medical Science (Beijing, China). All the experimental mice were housed under specific-pathogen-free conditions and fed normal chow and water ad libitum at Laboratory Animal Center of Institute of Biophysics, Chinese Academy of Sciences (Beijing, China).

All animal experiments were approved by the Biomedical Research Ethics Committee of the Institute of Biophysics, Chinese Academy of Sciences according to ‘‘Regulations for the Administration of Affairs Concerning Experimental Animals’’ (approved by the State Council on October 31, 1988). The animal experiments were performed in compliance with the Guidelines for the Care and Use of Laboratory Animals (Ministry of Science and Technology, NO. 398, 2006).

### Commercial antibodies and reagents

Anti-His Tag, anti-GST Tag and anti-myc Tag antibodies were from Sigma. Anti-pY418-Src, anti-Src, anti-p-ERK and anti-ERK antibodies were from Cell Signaling Technology. Anti-GAPDH was from Abcam. Anti-Na^+^/K^+^-ATPase α1 subunit was from Santa Cruz. The secondary antibodies of donkey anti-goat Alexa Fluor 555 and donkey anti-mouse Alexa Fluor 488 were from Invitrogen. The secondary antibody of HRP-conjugated goat anti-mouse or rabbit IgG was from GE Healthcare. All commercial antibodies were used according to the manufacturer’s instructions.

All chemicals were obtained from Sigma, and all cell culture media were purchased from Gibco. PP2, a Src kinase inhibitor, was from Calbiochem. U0126, an ERK kinase inhibitor, was from Cell Signaling Technology. G418 used in establishing stable transfectants was from Invitrogen.

### Cells, transfection and stable transfectants establishment

All cells were maintained at 37°C with 5% CO_2_. Human Hep3B cells were obtained from the ATCC and cultured in MEM supplemented with 10% fetal calf serum (FCS). Human MHCC97H cells were bought from Bicleaf Biotechnology Company (Shanghai, China) and cultured in DMEM supplemented with 10% FCS. Human HepG2, SNU449, Huh-7 and SMMC7721 cells were kindly gifted from Dr. Mingzhou Guo (Department of Gastroenterology and Hepatology; Chinese PLA General Hospital; Beijing, China) and were cultured in RPMI 1640 medium with 10% FCS. Fugene HD-mediated transfection was used according to the manufacturer’s instructions (Roche). Stable transfectants of SMMC7721-FXYD6 and SMMC7721-mock were established in the presence of 2 mg/mL G418.

### FACS (fluorescence activated cell sorting) analysis

1 × 10^5^ cells were stained with FD10 (2 μg/mL) for 1 h at 4°C and followed by Alexa Fluor 488-conjugated anti-mouse secondary antibody for 45 min at 4°C. The stained cells were analyzed for green fluorescence (FL1) with a FACSCalibur (Becton Dickinson).

### Surface plasmon resonance (SPR)

SPR experiments were performed using the Biacore T100 system (GE Healthcare) and the binding analyses were carried out at room temperature in 1× Phosphate Buffered Saline (PBS) with 0.05% Tween 20. FD10 was immobilized to the CM5 sensor chip (GE Healthcare) via standard N-hydroxysuccinimide and N-ethyl-N-(dimethylaminopropyl) carbodiimide activation. After immobilization, neutralization was done with 1.0 mol/L ethanolamine. The second flow channel on the same chip was activated/neutralized in a similar way and used as a negative channel. Increasing concentrations of purified FXYD6 protein were injected over the flow channels. FXYD6 was analyzed at 5 different concentrations, ranging from 0.15–12.15 μmol/L. The kinetic and equilibrium parameters (*K*_*D*_) values were calculated by fitting the raw sensorgram with the 1:1 drifting baseline-binding model by using Biacore T100 evaluation software.

### The commercial human tissue array

The use of human tissue assay was with informed consent and was approved by the Ethics Committee of the Institute of Biophysics, Chinese Academy of Science. This array (Aomei Biotechnology Co. Xi’an, China) includes a total of 397 human tissue specimens from different persons as following: 91 liver tissues (56 HCC versus 35 normal liver), 23 thyroid tissues (17 cancerous versus 6 normal), 19 prostate tissues (13 cancerous versus 6 normal), 23 colon tissues (17 cancerous versus 6 normal), 24 brain tissues (17 cancerous versus 7 normal), 25 uterus tissues (20 cancerous versus 5 normal), 21 bladder tissues (20 cancerous versus 1 normal), 36 ovarian tissues (30 cancerous versus 6 normal), 38 breast tissues (29 cancerous versus 9 normal), 27 testis tissues (21 cancerous versus 6 normal), 26 lung tissues (25 cancerous versus 1 normal), 23 stomach tissues (18 cancerous versus 5 normal), 21 pancreas tissues (15 cancerous versus 6 normal).

### Immunohistochemistry

For DAB staining, the commercial human tissue array was deparaffinized and stained firstly with FD10 (1 μg/mL), then incubated with Biotin-conjugated anti-mouse secondary antibody (ZSGB-BIO) and horseradish peroxidase-conjugated streptavidin (Thermo Fisher). The binding was detected by DAB solution (ZSGB-BIO). The tissues were then counter stained using hematoxylin (ZSGB-BIO). Images were taken with an OLYMPUS BX51 microscope with an UPlanFL N digital camera.

For immunohisto-fluorescence, the commercial human tissue array (AOMEI) was deparaffinized and co-stained with both anti-FXYD6 mAb FD10 (1 μg/mL) and anti-Na^+^/K^+^-ATPase α1 subunit antibody, followed by fluorescent-labeled secondary antibody. Nuclei were stained with DAPI. Pictures were taken with a confocal laser scanning microscope (Olympus FV1000) with an Olympus IX81 digital camera.

### The scoring algorithms of the immunohistochemistry

As scoring algorithms of the FXYD6 immunohistochemistry have not been optimized and standardized, we interpreted the cytoplasmic and nuclear staining collectively as listed in Table S1. The German semi-quantitative scoring system (Han et al., 2009) was adopted with some modification for scoring the results. Every human tissue specimen was given a score according to the intensity of the nuclear and cytoplasmic staining (no staining = 0; weak staining = 1; moderate staining = 2; strong staining = 3). The extent of stained cells was given as below: 0% = 0, 1%–10% = 1, 11%–50% = 2, 51%–80% = 3, and 81%–100% = 4. The final immuno-reactive score was determined by multiplying the intensity and extent of positivity scores of stained cells, with a minimum score of 0 and a maximum score of 12.

The threshold for differentiating between final positive and negative immunostaining was set at 4 for interpretation. A negative staining was classified as having an immunostaining score of 0–3; whereas a positive staining was classified as having an immunostaining score of 4–12 (at least moderately positive in at least 11%–50% of cells). Furthermore, a score of 4–6 is considered as low FXYD6 expression; whereas score of 7–9 means moderate expression and score of 10–12 means high expression.

### RNA interference

SiRNA against FXYD6 targeting its non-coding region and negative control siRNA (siRNA-nc) were from Invitrogen. The sequences of the siRNAs are as following. SiRNA-FXYD6: forward, 5′-CUGGCAGGCAAUAGUUGAAdTdT-3′; reverse, 5′-UUCAACUAUUGCCUGCCAGdTdT-3′; siRNA-nc: forward, 5′-CUUCAGCCUCAGCUUGCCGdTdT-3′; reverse, 5′-CGGCAAGCUGACCCUGAAGdTdT-3. The siRNA targeting FXYD6 does not affect the ectogenic FXYD6. For siRNA transfections, cells were transfected with 50 nmol/L siRNA and then incubated for at least 36 h at 37°C and 5% CO2.

### Cell proliferation analysis

After the appropriate treatments, cells were trypsinized and seeded in 96-well plates at a density of 4 × 10^3^ cells/well in 100 μL of complete medium (or containing 2 μmol/L PP2 or 5 μmol/L U0126 in the inhibition assay, or 100 μg/mL FD10 in the antibody blocking assay) and cultured for 24, 48, 72 and 96 h. After 2 h incubation with 10 μL of CCK-8 (Cell Counting Kit-8, DOJINDO), the optical density was measured at 450 nm.

### Cell migration analysis

Cell migration was analyzed using the Boyden chamber assay (8-μm pore size; Corning). After the appropriate treatments, cells were seeded in the upper chamber at a density of 5 × 10^3^ cells/well in 100 μL of serum-free medium (or containing 2 μmol/L PP2 or 5 μmol/L U0126 in the inhibition assay, or 100 μg/mL FD10 in the antibody blocking assay). 200 μL complete medium was added to the lower chamber. After culture for 7 h at 37°C, cells remaining at the upper surface of the membrane were removed using a swab. The cells, migrated to the lower membrane surface, are representative of the migrated cells. After fixation with 4% paraformaldehyde and staining with 0.1% crystal violet solution, the cells passed through the filter were counted and plotted as the number of migrated cells.

### Co-immunoprecipitation

Cells were lysed in culture dishes by adding 0.5 mL of ice-cold RIPA (radioimmunoprecipitation assay) lysis buffer for 45 min. The supernatants were collected by centrifugation at 12,000 *g* for 15 min at 4°C and then precleared with the protein G PLUS-Agarose beads (Santa Cruz) to remove the non-specific protein G-bounded proteins. The cleared lysate was then incubated with FD10 (5 μg/mL) at 4°C overnight, followed by incubation with the protein G PLUS-Agarose beads for 4 h. Immunoprecipitates were washed 3 times with lysis buffer and then analyzed by immunoblotting.

### Immunofluorescence

Cells were seeded on slides cultured in 6-well plates. After various treatments, the cells were washed with PBS, fixed in acetone/methanol (1:1) for 30 s, permeabilized with 0.1% Triton X-100, blocked with 5% normal donkey serum for 60 min at 37°C, and then incubated with anti-FXYD6 (FD10, 1 μg/mL) and anti-Na^+^/K^+^-ATPase α1 antibodies for 1 h. Then incubation with fluorescent-labeled secondary Abs for 45 min at 37°C, and nuclei were stained with DAPI. Confocal laser scanning microscope (Olympus FLUOVIEW FV1000) with an Olympus IX81 digital camera was used for photos collection.

### Xenografted tumor formation in nude mice

Female 4-week-old BALB/c nude mice were injected with 5 × 10^6^ SMMC7721-mock or SMMC7721-FXYD6 cells subcutaneously into the backs (6 mice per group). For antibody treatment, xenografts of human tumor cell lines were produced by injecting 1 × 10^6^ HepG2 cells subcutaneously into the backs of the mice. When tumors reached a diameter of 0.5–0.6 cm, the mice were grouped (5 mice per group) and intraperitoneally injected with mIgG or purified FD10 at a dose of 200 μg per mouse twice per week. Tumor size was measured twice per week and tumor volume was determined according to the following equation: tumor size = width^2^ × length × (1/2).

### Statistical analysis

All experiments were conducted in triplicate. Data are shown as mean ± SEM. The difference of FXYD6 expression levels in human tissues was analyzed with a χ^2^ test. Statistical differences were determined by unpaired Student’s *t* tests; except the statistical differences of the levels of the pY418-Src, p-ERK and Na^+^/K^+^-ATPase α1 subunit were determined by paired Student’s *t* tests. *P* < 0.05 was considered statistical significance.

## Electronic supplementary material

Below is the link to the electronic supplementary material.

Supplementary material 1 (PDF 297 kb)

## Abbreviations

CCK-8: Cell Counting Kit-8
co-IP: co-immunoprecitation
ELISA: enzyme-linked immunosorbent assay
FACS: fluorescence activated cell sorting
FCS: fetal calf serum
FXYD6: FXYD domain containing ion transport regulator 6
HCC: hepatocellular carcinoma
*K*_*D*_: equilibrium dissociation constant
mAb: monoclonal antibody
mIgG: normal mouse IgG
PBS: phosphate buffered saline
RIPA: radioimmunoprecipitation assay
siRNA-nc: negative control siRNA
SPR: surface plasmon resonance
