# Supplementary material for: FXYD6: a novel therapeutic target toward hepatocellular carcinoma
Source: Protein Cell. 2014 Apr 10;5(7):532–43. doi: 10.1007/s13238-014-0045-0 (PMC4085285; doi:10.1007/s13238-014-0045-0)
Supplement: Supplementary file 1 — Supplementary material 1 (PDF 297 kb) [file 13238_2014_45_MOESM1_ESM.pdf]

Protein & Cell

Supplemental Material

FXYD6: a novel therapeutic target toward hepatocellular carcinoma

Qian Gao, Xiongfei Chen, Hongxia Duan, Zhaoqing Wang, Jing Feng, Dongling Yang, Lina Song, Ningxin Zhou* and Xiyun Yan*

* Correspondence should be addressed to.

Professor Ningxin Zhou, Institute of Hepatobiliary Gastrointestinal Disease, General Hospital of PLA Second Artillery, 16 Xinjiekouwai Avenue, Xicheng, Beijing 100088, China. E-mail: zhounx301@163.com.

Professor Xiyun Yan, Institute of Biophysics, Chinese Academy of Sciences, 15 Datun Road, Beijing 100101, China. Email: yanxy@ibp.ac.cn. Fax: 086-10-64888584

Inventory of Supplemental Material:

1. Figure S1, Related to Figure 3. Co-localization of FXYD6 and Na^+/ K^+-ATPase in the HCC tissues.
2. Table S1, Related to Table 1. The Scores of Immunohistochemistry Staining.

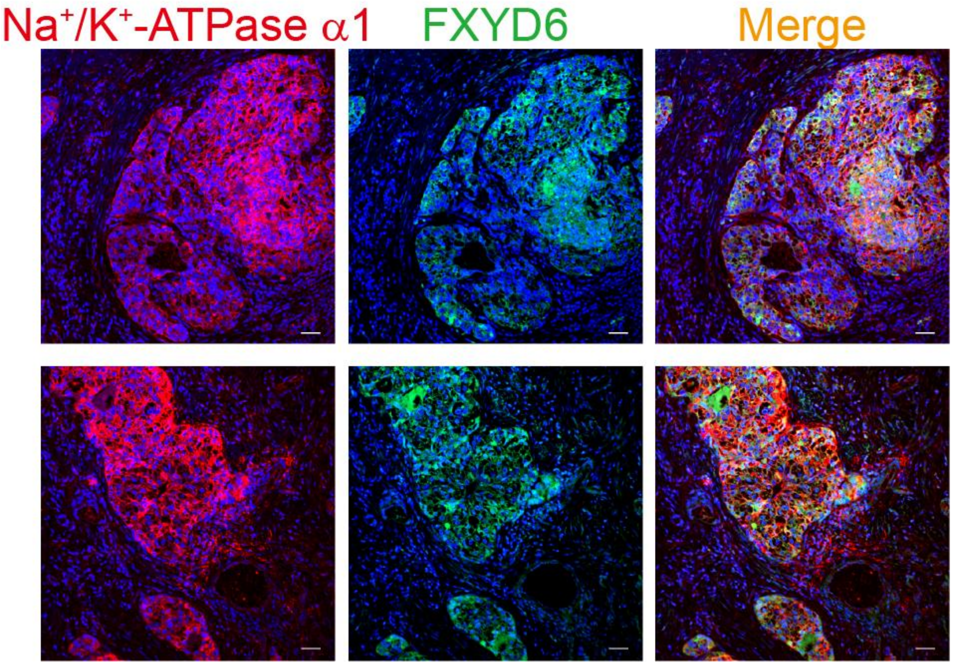

Figure S1. Co-localization of FXYD6 and Na^{+}/ K^{+}-ATPase in the HCC tissues.

Two hepatocellular carcinoma tissues from different patients were used for double immunostaining with anti-FXYD6 antibody (green) and anti-Na^{+}/K^{+}-ATPase α1 (red). The bar represents 40 μm.

Table S1. The Scores of Immunohistochemistry Staining

**Type of tissues**		**Total**	**Positive (Score 4-12)**	**Score 0-3**	**Score 4-6**	**Score 7-9**	**Score 10-12**
Liver	Hepatocellular carcinoma	56	54	2	8	27	19
	Normal tissue	35	11	24	7	1	3
Thyroid	Carcinoma	17	17	0	3	5	9
	Normal tissue	6	2	4	2	0	0
Prostate	Adenocarcinoma	13	9	4	2	5	2
	Normal tissue	6	1	5	1	0	0
Colon	Adenocarcinoma	17	17	0	0	4	13
	Normal tissue	6	4	2	0	0	4
Cerebrum	Astrocytoma	17	17	0	12	4	1
	Normal tissue	7	7	0	3	3	1
Uterus	Adenocarcinoma	20	20	0	6	4	10
	Normal tissue	5	5	0	0	1	4
Bladder	Transitional cell carcinoma	20	18	2	2	3	13
	Normal tissue	1	1	0	0	0	1
Ovary	Carcinoma	30	20	10	7	7	6
	Normal tissue	6	4	2	1	1	2
Breast	Carcinoma	29	18	11	9	9	0
	Normal tissue	9	6	3	1	3	2
Testis	Seminoma	21	12	9	5	6	1
	Normal tissue	6	5	1	3	1	1
Lung	Carcinoma	25	14	11	11	3	0
	Normal tissue	1	1	0	1	0	0
Stomach	Adenocarcinoma	18	9	9	4	4	1
	Normal tissue	5	3	2	1	2	0
Pancreas	Adenocarcinoma	15	5	10	2	2	1
	Normal tissue	6	3	3	1	2	0
